# Six-Month Outcomes of a Theory- and Technology-Enhanced Physical Activity Intervention for Latina Women (Pasos Hacia La Salud II): Randomized Controlled Trial

**DOI:** 10.2196/51708

**Published:** 2024-06-06

**Authors:** Lauren Connell Bohlen, Shira I Dunsiger, Tayla von Ash, Britta A Larsen, Dori Pekmezi, Becky Marquez, Tanya J Benitez, Andrea Mendoza-Vasconez, Sheri J Hartman, David M Williams, Bess H Marcus

**Affiliations:** 1 Center for Health Promotion and Health Equity Department of Behavioral and Social Sciences Brown University School of Public Health Providence, RI United States; 2 Herbert Wertheim School of Public Health and Human Longevity Science University of California San Diego La Jolla, CA United States; 3 Department of Health Behavior The University of Alabama at Birmingham School of Public Health Birmingham, AL United States; 4 Herbert Wertheim School of Public Health and Human Longevity Science Moores Cancer Center University of California San Diego La Jolla, CA United States

**Keywords:** digital health, web-based intervention, exercise, social support, behavior change intervention, support, Latina women, women, Latina, physical activity, barrier, aerobic, remote intervention, text message, behavior change, behavior

## Abstract

**Background:**

More than half (55%) of Latina women do not meet aerobic physical activity (PA) guidelines, and frequently cite time, childcare, and transportation as barriers to PA. In addition to linguistic adaptations for this population, successful PA interventions for Latina women addressed these barriers through remote intervention delivery approaches (eg, mail, phone, or web delivery).

**Objective:**

We aimed to evaluate 6-month outcomes of a randomized trial comparing a Spanish-language, individually tailored, web-delivered PA intervention (original) to an enhanced version with text messages and additional features (enhanced). Further, we evaluated if increases in PA at 6 months were moderated by baseline activity status.

**Methods:**

In total, 195 Latina women aged 18-65 years participated in a trial comparing the efficacy of the enhanced versus original interventions at initiating PA behavior change. We examined minutes per week of accelerometer-measured PA in the enhanced versus original arms, and the proportion of each arm meeting aerobic PA guidelines (150 min/wk at 6 mo). For moderator analyses, participants were classified as inactive (0 min/wk) or low active (1-90 min/wk) at baseline, measured via the 7 Day Physical Activity Recall interview.

**Results:**

PA increased from 19.7 (SD 47.9) minutes per week at baseline to 46.9 (SD 66.2) minutes per week at 6 months in the enhanced arm versus 20.6 (SD 42.7) minutes per week to 42.9 (SD 78.2) minutes per week in the original arm (*P*=.78). Overall, 30% (31/103) of the enhanced group met aerobic PA guidelines at 6 months, compared to 21% (19/92) of the original group (odds ratio [OR] 1.75, 95% CI 0.87-3.55). Baseline PA (inactive vs low active) moderated treatment effects on PA. For inactive participants, there were no group differences at 6 months (b=7.1; SE 22.8; *P*=.75), while low-active participants increased more in enhanced than original (b=72.5; SE 27.9; *P*=.01). For low-active participants, 45% (46/103) of the enhanced group met PA guidelines at 6 months, versus 20% (18/92) of the original arm (OR 3.29, 95% CI 1.05-11.31). For inactive participants, there were no group differences (25/103, 24% vs n=19/92, 21% for enhanced vs original, respectively; OR 1.28, 95% CI 0.54-3.06).

**Conclusions:**

Intervention effects were conditional on baseline PA. For low-active Latina women, the enhanced intervention was more effective at increasing PA. Additional tailored intervention enhancements may be necessary to increase PA for inactive Latina women.

**Trial Registration:**

ClinicalTrials.gov NCT03491592; https://www.clinicaltrials.gov/study/NCT03491592

**International Registered Report Identifier (IRRID):**

RR2-10.1186/s13063-022-06575-4

## Introduction

Rates of inactivity-related conditions (overweight, obesity, or type 2 diabetes) are both higher and growing faster among Latina women than in non-Latina White women [1,2], and it is projected that more than half of all Latina women will eventually develop type 2 diabetes or hypertension, or both [3,4]. There is robust evidence that regular physical activity (PA) can help prevent obesity, type 2 diabetes, and hypertension [5-7]. Thus, effective PA interventions that have the potential for widespread dissemination are needed for this at-risk population.

More than half (55%) of Latina women do not meet aerobic PA guidelines [8], and frequently cite barriers to PA such as childcare, transportation, and available time [9-11]. Moreover, Latina women report numerous social, environmental, economic, linguistic, and cultural factors that limit their access to PA programs and resources [12]. Successful PA interventions for Latina women have made linguistic adaptations for this population and further sought to address these barriers through remote intervention delivery approaches (eg, mail, phone, or web delivery) [13,14].

The use of technology-based tools and resources to improve access to health-related interventions shows great promise as a low-cost solution for targeting disproportionately affected populations [15]. There is near universal use of the internet among Latina women (approximately 92%), with access to the internet primarily mediated through smartphones [16]. Yet, few PA interventions use technology-based tools to conduct interventions in Latina populations. Noting the rapid growth of internet use among the Latino population [17], and building upon the success of our research team’s evidence-based print intervention [18], we previously conducted formative research with Latina women to develop a theory-based and web-based PA intervention [17]. The results of our initial web-based PA intervention showed that participants achieved significant PA improvements at 6 and 12 months [14,19]; however, participants were on average still well below the national PA guidelines for aerobic activity, suggesting that a more intensive internet-based approach may be necessary to help Latina women reach and sustain health-enhancing PA levels.

As such, a series of technology and theory-supported adaptations were made to the original intervention, including optimization of the website for mobile viewing, text messages to increase engagement with the website, and further targeting the intervention toward changing key social cognitive theory [20] constructs (eg, self-efficacy, enjoyment, and social support).

This study builds upon our previous intervention and formative research with Latina women to test the original individually tailored, Spanish-language, web-based Pasos Hacia La Salud Intervention (original intervention) to a text-message and theory-enhanced version (enhanced intervention) [14,19]. This paper aims to evaluate changes in moderate to vigorous physical activity (MVPA) in the original versus enhanced conditions from baseline to 6 months, as described in this study’s protocol paper [21]. A secondary aim of this paper is to explore differences in activity increases in individuals in the contemplation stage versus the action transtheoretical model (TTM) [22] stage of change at baseline; that is, to compare individuals who were doing no activity (0 min/wk) to those doing some amount of activity (>0 min/wk), to better understand who is best aided by the enhancements.

## Methods

### Study Design

This 6-month randomized controlled trial tested the relative efficacy of an enhanced versus original evidence- and theory-based, Spanish-language PA intervention website on changes in MVPA at the end of the active intervention period (6 mo). The study was conducted at Brown University and registered as a clinical trial (NCT03491592).

### Ethical Considerations

All study procedures were approved by the Brown University Institutional Review Board (IRB#: 00000556) and described in detail in a prior report [21]. All participants provided written informed consent.

### Participants

Participants included those who self-identified as female and Latina. For inclusion, participants were required to be aged 18-65 years, and engaging in less than 60 minutes per week of self-reported leisure-time MVPA. To be eligible, participants needed to score more than 16 on the Short Test of Functional Health Literacy in Adults, to determine whether participants have adequate literacy levels to read study materials in Spanish [23-25]. This measure has high internal validity (>0.95 in either language) and strong positive associations with education. Additional eligibility criteria included regular access to text-message compatible and internet-connected devices through home, work, or their community. Exclusion criteria included any serious medical condition that could make unsupervised PA unsafe (based on the modified Physical Activity Readiness Questionnaire) [26], including a history of coronary heart disease (history of myocardial infarction or symptoms of angina), stroke, or orthopedic conditions which limit mobility; other exclusion criteria were hospitalization due to a psychiatric disorder in the past 3 years, BMI above 45 kg/m^2^, current or planned pregnancy, or moving from the area within this study’s time frame.

### Interventions

#### Original Intervention: Pasos Hacia La Salud I

In the original web-based intervention program, participants attend an in-person or Zoom-based (Zoom Video Communications, Qumu Corporation) baseline session led by trained bilingual or bicultural interventionists to be oriented to the intervention website and set incremental goals. The interventionist guides participants in goal setting (eg, setting a personalized weekly MVPA goal), and instructs them on how to progressively increase their goals to build toward meeting national PA guidelines (ie, graded tasks). The goal-setting session is based on principles of motivational interviewing and also teaches problem-solving skills so participants can adjust plans when necessary. In addition to this baseline goal-setting session, participants complete goal-setting sessions at month 1, and during assessment follow-up visits. Participants were encouraged to accumulate moderate-intensity PA on a majority of the days of the week and recommended to achieve a goal of 150 minutes of MVPA by the 6-month visit. Participants were not required to accumulate MVPA in 10-minute bouts.

Web-based tools to help participants increase their MVPA include (1) self-monitoring of behavior (eg, calendars to log weekly activity), (2) discrepancy between current behavior and goal (eg, calendars display weekly goals alongside weekly activity), (3) social support (eg, a discussion board where participants can interact), (4) instruction about how to be physically active (eg, information about local PA resources and places to be active), (5) problem solving (eg, tips for overcoming common PA barriers; neighborhood safety, and caregiving responsibilities), (6) motivational stage-matched PA manuals based on the TTM [17,22], and (7) individually tailored feedback based on their responses to online questionnaires assessing self-efficacy for PA, processes of change for PA, and social support. Further details about the tailored intervention content are described elsewhere [21]. Participants are encouraged by staff to regularly access the website, log their PA on the website, and receive a tip of the week. Participants are also prompted to complete online surveys to tailor their intervention content every month.

#### Enhanced Intervention: Pasos Hacia La Salud II

In addition to all of the intervention components in the original intervention described above, Pasos II includes the following enhancements: (1) text messages, (2) additional phone calls, and (3) additional data-driven content and website features that require user participation. In previous trials, the extent to which the Pasos I intervention increased PA behavior was associated with greater use of the website (ie, logging in more frequently, spending more time on the website, and use of specific dynamic features). Previous trials also demonstrated that the social cognitive theory [20] constructs, particularly self-efficacy, enjoyment, and social support, were predictive of PA behavior in this population. Thus, the enhancements to the original intervention were designed to increase participant engagement with the website and further target these constructs.

For 6 months after randomization, participants received daily text messages (in Spanish), which were personalized, and targeted self-efficacy, enjoyment, and social support (eg, “You can combine fun and social time with exercise.” “Do you enjoy dancing? Take a weekly dance class of flamenco, salsa or tango. Be adventurous!”), along with reminders to use the tracking features on the website (eg, What would you like your exercise goal to be this week? Login to the website to enter it on the calendar! Login to the website to enter it on the calendar!). Participants also received additional phone calls at 2 and 3 months to check on their progress and discuss any barriers to them achieving their PA goals.

To further promote website engagement, participants were provided with nonmonetary, material incentives for their website use (Multimedia Appendix 1). Specifically, participants could earn points to receive items like water bottles and phone cases. Participants earned points through logging on to this study’s website, and using features on the website such as posting a PA goal, completing a questionnaire, and other features which were associated with the largest change in PA in the original study. In weeks that participants self-recorded at least 150 minutes of PA, they were awarded a web-based “medal.” These medals could be collected cumulatively and were displayed for participants to view in their goal-setting tab on the website. Earning this medal meant that participants were part of the “150 Minute Club” and they got to see their name posted on the “150 Minute Club” board. All participants in the enhanced arm could view the names of members currently, and recurrently in the “150 Minute Club.” To enable additional opportunities for engaging with the intervention content, the motivational stage-matched manuals were made available in both audio and written formats.

To further enhance social support for PA, a discussion board was used to facilitate community meetups among participants. Research staff would post details about free and low-cost PA events in the area so that participants could meet up, discuss coordinating attendance with others, overcome barriers to PA, and motivate each other to attend these events.

### Procedures

#### Recruitment

Community-based strategies were used to recruit participants including a Facebook (Meta) page, internet-based advertising, in-person recruitment at community events and organizations, advertisements in Spanish language newspapers, radio stations and television advertising, and flyers posted in the community. Participants were primarily recruited from in and around Providence County in Rhode Island.

#### Baseline Sessions

Following recruitment, eligible participants met with bilingual, bicultural staff for an orientation session, who outlined study requirements, potential risks, and benefits, and completed the informed consent process with participants. Participants were given an accelerometer-based device (ActiGraph wGT3X-BT; ActiGraph, LLC) to wear for 7 continuous days before returning for their randomization visit. After participants completed all baseline measures, they were randomly allocated to 1 of the 2 (original or enhanced) PA interventions.

#### Randomization

Participants were randomized to 1 of 2 conditions using a permuted block randomization scheme designed by this study’s biostatistician, which was stratified by baseline stage of change for PA Behavior according to the TTM; with most participants being in contemplation (intending to increase PA but not currently doing any activity) or action (doing some activity, but not meeting national activity guidelines). The study biostatistician created the random allocation sequence, and all staff were blinded to the allocation sequence. Outcome assessment staff are blind to the intervention condition, and intervention staff and participants are not blind to the participant intervention condition.

### Measures

#### Overview

Following baseline assessments, participants’ MVPA was assessed again at 6 months. Participants received a questionnaire package and an accelerometer-based device approximately 1 week before the assessment visit. All measures listed below were given at baseline and month 6, available in Spanish, and have been used in our previous studies [14,17-19,27].

#### Primary Outcome

The primary outcome was the total weekly minutes of MVPA measured via an accelerometer (ActiGraph wGT3X-BT) [28], which measures the movement and intensity of PA. Participants were instructed to wear the ActiGraph wGT3X-BT accelerometer-based device on their hip during waking hours for 7 consecutive days. Participants wore the device on weekdays and weekends, and for example, if a participant started wearing the device on Tuesday they would be asked to wear the device during waking hours until the end of the day on the following Monday. They also were instructed to write down the dates and times they put on and took off the accelerometer-based device. Per standard procedures, the minimum acceptable wear time was 5 days with at least 600 minutes daily or 4 days with at least 3000 minutes total. Daily and weekly minutes of MVPA were calculated with ActiLife (ActiGraph, LLC) software, using a minimum cutoff point of 1952 [29] to define moderate-intensity PA and a minimum activity bout of 10 minutes, for consistency with the original Pasos Hacia La Salud study [14,19].

#### Secondary Outcome

To provide additional detail about PA, and to capture more information about the types of activities, participants completed an interview to self-report their total weekly MVPA as a secondary outcome measure. The 7-day Physical Activity Recall (PAR) interview [30,31] provides an estimate of the total weekly minutes of PA. The 7-day PAR has repeatedly shown acceptable internal consistency, reliability, and concurrent validity with objective measures of MVPA [32-34], along with sensitivity to changes [33,34] in both moderate and intensive levels of PA [35,36]. Additionally, the 7-day PAR has demonstrated test-retest reliability among Latino participants [37]. The 7-day PAR collects data on any physical activity that is completed during the week. The interviewer would ask the participant to categorize the purpose of the activity into (1) leisure, (2) occupational, (3) housework, and (4) transport. All activities were collected regardless of their purpose, however, activities designated as “housework,” “occupational,” or “transport” were removed from the total number of minutes when determining eligibility for participation in this study.

#### Baseline PA

We evaluated baseline PA, as measured via self-report, as a potential moderator of intervention effects on outcomes at 6 months. To evaluate baseline PA as a moderator we dichotomized baseline PA levels based on whether participants were doing any activity at all at baseline (low-active) versus those doing no activity at all at baseline (inactive or 0 minutes of baseline MVPA).

### Data Analyses

We assessed potential between-group differences in baseline characteristics (demographics and activity level) using graphical methods, *t* tests for continuous variables, and *χ*^2^ tests for categorical variables. We examined the effects of the enhanced versus the original intervention at the end of the active intervention period on our primary outcome: minutes per week of objectively measured MVPA at 6 months using a generalized linear model with the identity link function. We controlled for the baseline value of the outcome, accelerometer wear time as well as a time-varying indicator of before vs during pandemic time). A similar series of regression models assessed between-group differences in the secondary outcome (self-reported MVPA). For all analyses involving self-reported MVPA, the total amount of weekly PA reported was based on all types of activity, thus including leisure, occupational, transport, and housework categories of MVPA.

As a subsequent step, given the high proportion of participants who were inactive at baseline (0 min/wk of self-reported MVPA), we examined the potential conditional effects of the intervention as a function of baseline activity level (dichotomized as inactive vs not). Effects were examined using similar models to those described previously: a generalized linear model that included the main effects of intervention (enhanced vs original), the potential moderator (indicator of being inactive at baseline), as well as the interaction between the two. Evidence of moderation exists if the coefficient of the interaction term is statistically different than zero. To assess whether the differences in the effects of treatment were a function of the amount of activity being reported, we ran a similar set of models with a continuous moderator (min/wk of MVPA at baseline), as opposed to the dichotomous version (no activity vs at least some). Analyses were performed with self-reported MVPA as the moderating variable and with accelerometer-measured MVPA as the moderating variable (continuous and dichotomous).

Next, using a generalized linear model, we examined the effects of the intervention on the proportion of participants meeting national PA guidelines for aerobic activity (defined as at least 150 min/wk of MVPA) at 6 months, measured by an accelerometer-based device. Models specified a logit link function and estimated odds ratios (OR and corresponding 95% CIs) as measures of effect. Models were run first, only specifying the main effect of treatment assignment (original vs enhanced intervention) and then conditional on baseline PA (inactive vs not).

All analyses were based on the intent to treat the sample, with all randomized participants included in the analysis. Details about power analyses conducted to determine the sample size are reported elsewhere [21]. Generalized linear models use a quasi-likelihood-based approach to estimation thus making use of all available data (intent-to-treat sample) without directly imputing missing values. Analyses were run in SAS (version 9.4; SAS Institute Inc) with a significance level set at 5% a priori.

## Results

### Sample Characteristics

**Figure 1:**
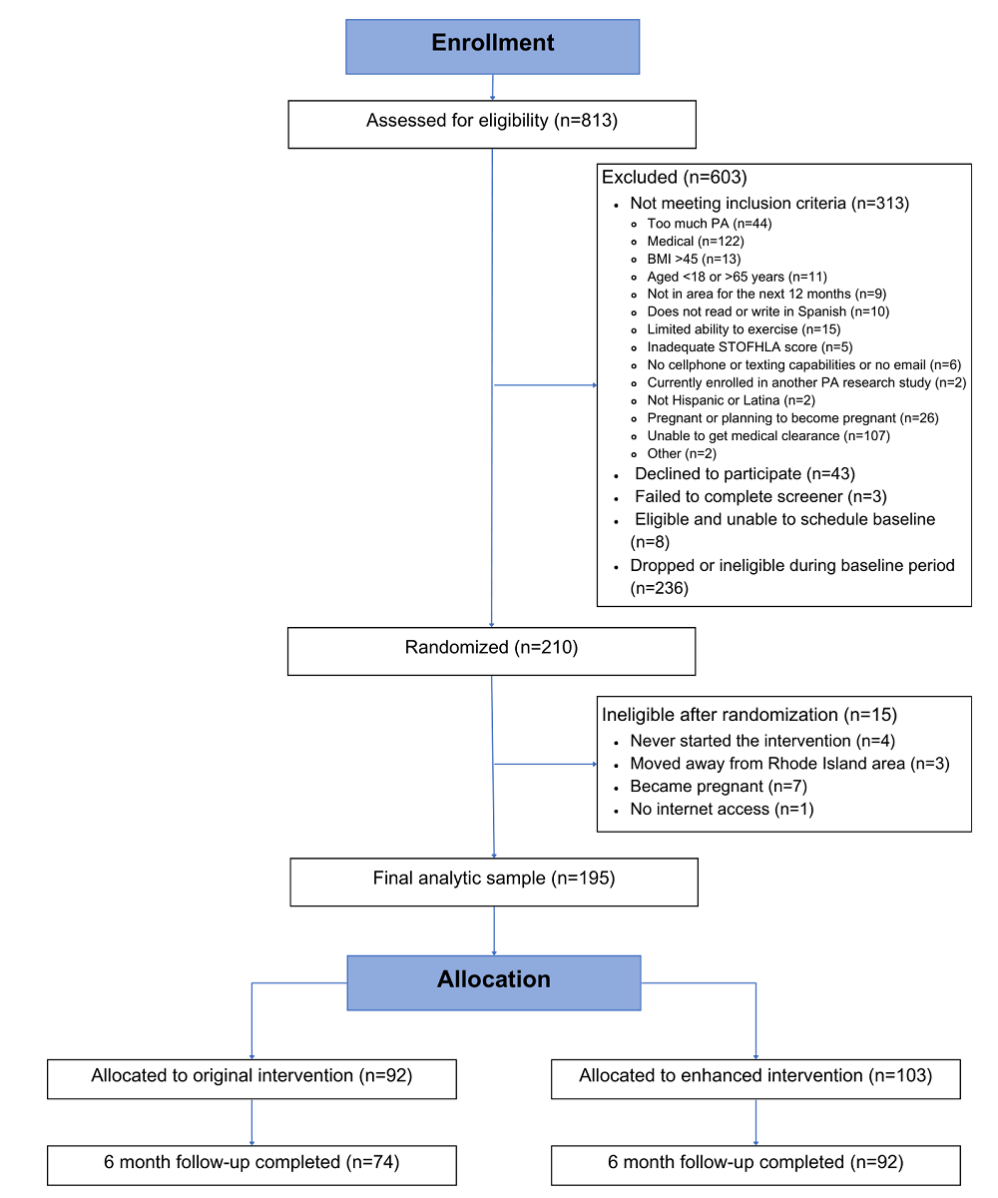
PASOS II month 6 CONSORT diagram. CONSORT: Consolidated Standards of Reporting Trials; PA: physical activity; STOFHLA: Short Test of Functional Health Literacy in Adults.

A full description of this study sample is presented in Table 1. The final sample included 195 participants randomized to the original PA intervention (n=92) or the enhanced intervention at baseline (n=103). Overall, participants were aged 43.31 (SD 10.29) years on average, predominantly Dominican (n=80 or 41%), 97.5% (n=190) of participants were first generation, with 62.4% (n=122) participants reporting at least a high school level education. Of note, 68.7% (n=134) of participants were inactive at baseline. There were no differences between intervention conditions at baseline for sociodemographic variables or PA level (*P*>.05). The flow of participants who were assessed for eligibility, randomized, and who attended follow-up visits are depicted in the CONSORT diagram (Figure 1). In the diagram, “completed” indicates those who attended the 6-month measurement visit and have data for the 7-day PAR. At 6 months, 133 participants completed the accelerometer-based device measurements, with no differences between conditions.

**Table 1:** Baseline characteristics of study sample by study arm (N=195)^a^.

Characteristics			Enhanced (n=103)		Original (n=92)		All (N=195)
Age (years), mean (SD)			42.93 (10.17)		43.73 (10.47)		43.31 (10.29)
**Country of origin^b^, n (%)**							
	Puerto Rico	11 (10.7)		16 (17)		27 (13.8)	
	Mexico (including Mexican American and Chicana women)	6 (5.8)		5 (5)		11 (5.6)	
	Cuba	0		0		0	
	Guatemala	14 (13.6)		13 (14)		27 (13.8)	
	Colombia	22 (21.4)		11 (12)		33 (16.9)	
	Dominica	39 (37.9)		41 (45)		80 (41)	
	Other	16 (15.5)		15 (16)		31 (15.9)	
**Race^c^, n (%)**							
	American Indian or Alaskan Native	5 (4.8)		1 (1)		6 (3.1)	
	Asian	1 (1)		0		1 (0.5)	
	Black or African American	14 (13.6)		12 (13)		26 (13.3)	
	Mixed	4 (3.9)		2 (2)		6 (3.1)	
	Native Hawaiian or other Pacific Islander	1 (1)		1 (1)		2 (1)	
	Other	26 (25.2)		16 (17)		42 (21.5)	
	White	31 (30.1)		39 (42)		70 (35.9)	
**Education^c^, n (%)**							
	<High school	36 (34.9)		34 (37)		70 (35.9)	
	High school graduate	14 (13.6)		8 (9)		22 (11.3)	
	Vocational or technical school	7 (7.1)		11 (12)		18 (9.2)	
	Some college	18 (17.5)		13 (14)		31 (15.9)	
	College graduate	22 (21.3)		18 (20)		40 (20.5)	
	Postgraduate	2 (1.9)		3 (3)		5 (2.6)	
**Employment status^c^, n (%)**							
	Unemployed	35 (34.7)		19 (21)		54 (27.7)	
	Employed full time	41 (40.6)		42 (46)		83 (42.6)	
	Employed part-time	25 (24.8)		25 (27)		255 (28.6)	
**Marital status^c^, n (%)**							
	Never married or lived with partner	19 (18.4)		15 (16)		34 (17.4)	
	Divorced	13 (12.6)		9 (10)		22 (11.3)	
	Separated	11 (10.7)		11 (12)		22 (11.3)	
	Widowed	3 (2.9)		4 (4)		7 (3.6)	
	Married	45 (43.7)		44 (48)		89 (45.6)	
	Living with partner	9 (8.7)		9 (10)		18 (9.2)	
BMI, mean (SD)			29.33 (6.59)		30.89 (6.30)		30.09 (6.48)
**Baseline MVPA^d^ (min/wk), mean (SD)**							
	Accelerometer-measured	19.70 (47.93)		20.57 (42.69)		20.11 (45.40)	
	Self-reported^e^	55.28 (262.07)		52.43 (150.27)		53.94 (216.11)	
Acculturation, STOFHLA^f^ score, mean (SD)			33.50 (3.70)		33.30 (3.34)		33.41 (3.53)

^a^Not all categories sum to 100% due to rounding of percentages.

^b^Participants could select more than one option in response to the question about their country of origin; thus, the numbers sum to more than the total for the respective treatment arm.

^c^Numbers for these categories do not sum to 100 due to missing data for some questions.

^d^MVPA: moderate to vigorous physical activity.

^e^Self-reported moderate to vigorous physical activity represents all types of physical activity in this table; eligibility was based on only leisure time physical activity.

^f^STOFHLA: Short Test of Functional Health Literacy in Adults.

### Changes in Physical Activity Over Time

Mean minutes per week of accelerometer-measured MVPA increased significantly in both groups, from 19.70 (SD 47.93, median 0) at baseline to 46.90 (SD 66.24, median 19.00) at 6 months among enhanced participants compared to 20.57 (SD 42.69, median 0) to 42.87 (SD 78.18, median 0) among those randomized to the original intervention. An adjusted model (adjusting for baseline MVPA and accelerometer wear time) did not indicate significant between-group differences at 6 months (*P*=.78).

The 7-day PAR interview findings were similar. Participants in the enhanced intervention condition increased self-reported MVPA from 57.88 (SD 260.25, median 0) minutes per week at baseline to 114.97 (SD 119.63, median 75) compared to the original intervention who reported an increase from 55.19 (SD 149.12, median 0) to 87.88 (SD 115.22, median 52). There were no differences between intervention conditions by self-reported MVPA at 6 months (*P*=.13). Thus, there was a significant increase from baseline to 6 months in MVPA in both groups, but this increase was not significantly different between treatment arms for either self-report or accelerometer-measured PA.

We found a moderating effect of self-reported baseline PA (inactive vs not) on both accelerometer-measured and self-reported MVPA at 6 months (controlling for baseline). For inactive participants, all participants reported 0 total minutes of self-reported MVPA at baseline.

**Figure 2:**
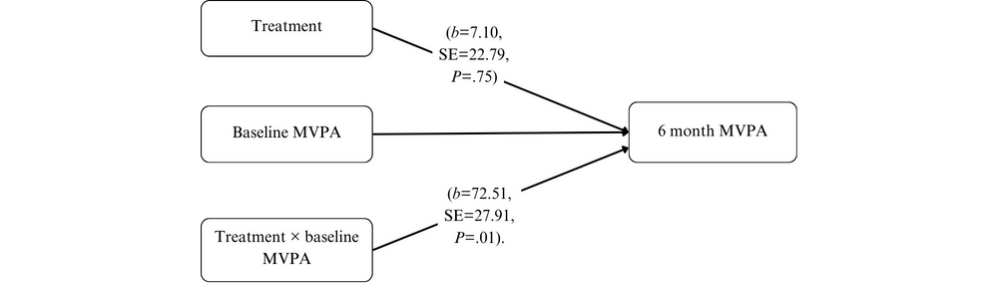
Path diagram depicting the results of the moderator analysis of unadjusted minutes per week of MVPA (self-reported MVPA) stratified by baseline self-reported MVPA. MVPA: moderate to vigorous physical activity.

For low-active participants, the median baseline level of MVPA was 48 minutes per week, with 25% (n*=*49) of the sample reporting 20 minutes per week or less. Among inactive participants, there was no significant effect of the enhanced intervention compared to the original intervention on self-reported MVPA (*b*=7.10; SE 22.79; *P*=.75). For those reporting some activity at baseline (low-active), significant effects were detected which favored the enhanced intervention (*b=*72.51; SE 27.91; *P*=.01). Similar findings were seen with accelerometer-measured MVPA, with significant effects of the enhanced intervention among those who reported at least some activity at baseline (*b*=21.51; SE 8.32; *P*=.02). Moderating effects were not detected based on the *amount* of activity being done at baseline (that is, the number of minutes), only on whether participants did some versus none (see Figure 2). There were no moderating effects of minutes per week of MVPA at baseline, suggesting intervention effects were not conditional on the quantity of PA at baseline, but just whether or not *any* was being completed. The findings were nearly identical when we examined the moderating variable of inactive versus low-active with accelerometer-measured MVPA, and the 6-month MVPA outcomes were self-reported or accelerometer-measured.

Looking across all participants, 30% (n*=*31) of participants in the enhanced intervention condition met national PA guidelines for aerobic activity at 6 months based on self-reported data, compared to 21% (n*=*19) of original intervention (OR 1.75, 95% CI 0.87-3.55). No participants met aerobic PA guidelines at baseline in either condition. Much like the continuous outcome for self-reported MVPA, for low-active participants, 45% (n*=*46) of participants in the enhanced intervention met national PA guidelines at 6 months compared to 20% (n*=*18) of original intervention participants (OR 3.29, 95% CI 1.05-11.31). For those who were inactive at baseline, there was no difference between arms in meeting national guidelines (n*=*25 vs n*=*19, 24% vs 21%) for enhanced versus original intervention (OR 1.28, 95% CI 0.54-3.06).

## Discussion

### Principal Findings

The objective of this paper was to evaluate whether the enhanced Pasos Hacia La Salud II intervention significantly increased PA, and helped more Latina women meet PA guidelines within 6 months, compared to the original intervention (Pasos I). The primary goal of this study was to evaluate whether there was a difference between the intervention conditions at reaching our primary end point: meeting national PA guidelines at 6 months. Overall, the results of this study indicate that both the enhanced and original intervention conditions helped Latina women increase their PA during the 6-month intervention. Both arms of the intervention appeared equally effective, with a significant increase in PA from baseline, yet there were no significant differences between groups in overall changes in minutes of activity or in meeting national guidelines.

However, the findings indicated that meeting aerobic PA guidelines and increasing PA differed for those participants who started the program engaging in some amount of MVPA at baseline, compared to those who started with no MVPA. For participants doing even a small amount of MVPA at baseline, the enhanced intervention was more effective at increasing the mean number of minutes per week of MVPA from baseline through 6 months, compared to the original condition. For those doing no activity at baseline, however, there were no differences in the intervention effects by treatment condition. Furthermore, the enhanced intervention resulted in more participants reaching the national aerobic PA guidelines within 6 months than the original intervention, only among low-active participants (ie, those who were doing some activity at baseline). Notably, this moderator effect was seen only for the dichotomous outcome (some vs no activity), not for baseline activity as a continuous outcome, suggesting an important difference between those who were doing some amount of MVPA albeit small (ie, 25-50 min/wk)—compared to those who had not yet begun to engage in any MVPA.

For the overall main effects, the findings are comparable to the original intervention study, which was conducted among a geographically and culturally different population of Latina women. In Pasos I, the participants were predominantly Mexican American (84.4%), and first-generation Americans (81.9%) recruited from the San Diego, California, area [14], whereas in Pasos II the participants were predominantly Dominican recruited from the Providence, Rhode Island, area and 97.5% were first generation. Despite differences in this study’s settings and study participants, in both settings, the original intervention increased minutes of PA at 6 months. In Pasos I, intervention participants increased their self-reported MVPA from 8 minutes per week to over 100 minutes per week [14], and in Pasos II, participants receiving the same intervention as those in Pasos I increased their self-reported PA from an average of 55 (SD 262.07) minutes at baseline to more than 85 minutes per week, and the enhanced intervention participants increased from 57 minutes at baseline to more than 114 minutes at 6 months. Future research can examine statistical comparisons of these 2 samples through integrative data analysis, which is beyond the scope of this paper. Of note, the intervention evaluated in both of these trials indicates generalizable efficacy for increasing MVPA in Latina women, in 2 different samples of Latina women, in 2 geographically different regions.

Few other studies have been conducted that evaluate the effects of a culturally tailored, technology-delivered PA intervention for Latina women. In a systematic review of culturally tailored, technology-delivered interventions for changing health behaviors, there is mixed support across health behaviors to recommend culturally adapting technology-delivered interventions [38]. Notably, for PA behavior, there is a small but significant effect that favors culturally tailored PA interventions from 5 previous randomized controlled trials. This review included the efficacy trial which tested the original Pasos intervention, and the addition of this study’s findings support the evidence in favor of a culturally tailored PA intervention for Latina women [38]. Additional studies evaluating PA interventions tailored to the Latino population detect similar results. For example, a previous study examined the effects of a PA intervention for Latina women in faith-based organizations. This study identified a marginal increase in self-reported MVPA and nonsignificant findings for accelerometer-measured PA behavior [39]. Although the findings are comparable, the key difference between this study and the faith-based culturally tailored PA intervention is that the latter requires substantial in-person involvement, and thus a large amount of staff time is required to implement an intensive in-person PA intervention. This difference in intervention delivery has important implications for the future scalability and dissemination of PA interventions for Latina women in real-world contexts, with constraints around staff- and participant time for in-person interventions.

The results of this study expand upon the generalizability of the findings for the original intervention by testing the effects among a geographically and culturally different population. However, the conditional effects of the enhanced intervention may not generalize to other Latina subgroups, Latino men, or other ethnic groups. For example, features specific to the enhanced arm such as the discussion board with opportunities for group meetups, text messages, and other content may not be as engaging or as motivating as it was for this group. Additionally, although the enhanced intervention added features to improve engagement with the intervention (by prompting the use of the website, and offering both audio and written versions of some materials), future research could further expand engagement with the program through the addition of infographics, video, or additional audio-based content.

While it is possible that the improvements in PA in this study could be seen as modest, with one-third of participants reaching the recommended PA levels in 6 months, it is important to note the distinction in the effects of the intervention for those who were doing any activity at all, versus those doing none. Nearly two-thirds of the sample were doing no PA at all at baseline. To motivate completely inactive individuals to become active may be the most difficult part of the behavior change process, compared to those who are low-active, which was reflected in our results. While both conditions significantly helped participants increase their minutes of MVPA per week, the intervention enhancements were most helpful for participants doing some activity at baseline, not for completely inactive participants. Specifically, the moderator findings in particular highlight that there are possibly differences in the effects of the enhanced versus original intervention for those who are contemplating change, versus those who have already started to change their MVPA behavior [22]. Previous research suggests that the underlying barrier to the stage of change progression is different for those who are in the contemplation stage (deciding whether to change) versus those who are in the preparation stage (insufficient beliefs about their abilities to successfully change) [40]. Thus, while the original intervention is known to increase self-efficacy [14], the intervention enhancements were designed to further support increases in self-efficacy, and promote engagement with the original intervention which may be 1 explanation for the moderating treatment effects. However, these enhancements may not be providing enough support in the decision-making process for inactive Latina women to become physically active. This could potentially be facilitated by using additional forms of technology.

For example, delivering the intervention via a smartphone app, wearables, or other technology-based tools may increase convenience and would enable the use of push notifications to nudge participants, which might be particularly beneficial for inactive individuals. To date, few other technology-delivered interventions have been tested that are culturally tailored to the Latina and Latino populations. Four other published studies used similar technology to Pasos I and Pasos II, using the internet, or text messages to increase MVPA [41]. Two other studies evaluated MVPA interventions specifically designed for the Latino population and used fitness wearables, or fitness-tracking mobile apps as part of the intervention [42,43]. These interventions resulted in significant increases in MVPA, and given that both were feasibility studies using smaller sample sizes, with less than 6 months follow-up [42,43], future research might evaluate whether more advanced fitness technology may help to augment the effects of the Pasos intervention. However, such interventions would likely exclude individuals without access to certain devices, including smartphones, having implications for addressing health disparities.

Lastly, this study identified changes in MVPA after 6 months, and previous MVPA interventions among Latina women have either evaluated shorter follow-up durations [41-43] or have failed to identify longer-term maintenance of intervention effects [19]. Thus, while this study identified that the enhanced intervention arm may lead to significantly greater increases in MVPA after an initial period for Latina women doing any MVPA at baseline, future research should evaluate whether (1) these findings persist beyond 6 months and (2) whether those who were inactive at baseline need either more support or more time (>6 mo) for the original or enhanced Pasos intervention to increase MVPA behavior.

The findings from this study indicate that an enhanced version of the web-delivered, individually tailored intervention for Spanish-speaking Latina women resulted in larger increases in PA compared to the original (Pasos I) intervention, but only among those who were low-active at baseline. Overall, the enhanced version of the intervention did not outperform the original version, and both versions of the intervention significantly increased MVPA in Latina women. The enhanced version of the website incorporated text messaging, contained dynamic features to encourage website use, and featured additional targeting of key theoretical constructs identified as important mechanisms of action in the prior study [14,44]. Future research should seek to better understand the extent to which additional tailoring, time, or intervention techniques may be needed for those who report doing no PA at all and seek to understand methods for the dissemination and implementation of the enhanced intervention for low-active Latina women.

## Appendix

Multimedia Appendix 1 Description of material incentives participants in the enhanced intervention condition could earn.

Multimedia Appendix 2 CONSORT eHEALTH checklist (V 1.6.1).

## Abbreviations

MVPA: moderate to vigorous physical activity
OR: odds ratio
PA: physical activity
PAR: Physical Activity Recall
TTM: transtheoretical model
