# Supplementary material for: Six-Month Outcomes of a Theory- and Technology-Enhanced Physical Activity Intervention for Latina Women (Pasos Hacia La Salud II): Randomized Controlled Trial
Source: J Med Internet Res. 2024 Jun 6;26:e51708. doi: 10.2196/51708 (PMC11190618; doi:10.2196/51708)
Supplement: Multimedia Appendix 1 [file jmir_v26i1e51708_app1.docx]

**Multimedia Appendix 1.** Description of material incentives participants in the enhanced intervention condition could earn***.***

Participants could receive incentives by earning points. Participants earned points for completing activities on the intervention website which would promote, and/or support their exercise behavior. Notably, participants did not earn points for completing certain amounts of exercise behavior, only for the act of self-monitoring their behavior.

Point Structure:

- 1 point for entering (logging into) the website - up to a max of 3 points per day
- 10 points for recording activity on the website, up to a max of 1 time a day
- 50 points for setting a goal, max of 1 time a week
- There were also bonus points that were randomly given for the same activities. Participants were eligible for bonus points if they completed any of the above three activities which would earn them points
- *Bonus Point Structure:*
  - 10 points - logging into the website
  - 25 points - registering activity on the website
  - 100 points setting goals

Earning Prizes:

Participants earned points which accumulated over time. Once they hit a certain point total they automatically received a prize. They did not pick and/or choose which prize, or how to ‘spend’ their points, and the points never re-set.

Examples of prizes included: water bottles, phone cases, notebooks, tote bags, t-shirts and sweatshirts, yoga mats, jump ropes, exercise phone case holder.
